# Integrated Transcriptomic and Metabolomic Analysis Reveals Biochar-Induced Enhancement of Growth and Secondary Metabolism in the Medicinal Plant *Echinacea purpurea*

**DOI:** 10.3390/ijms262311249

**Published:** 2025-11-21

**Authors:** Liru Tan, Ling Liu, Jun Liu, Yawen Fu, Ying Zhang, Caixia Sun, Yulan Zhang

**Affiliations:** 1 Key Laboratory of Bioresource Research and Development of Liaoning Province, College of Life and Health Sciences, Northeastern University, Shenyang 110169, China; tanliru@mails.neu.edu.cn (L.T.); 2301507@stu.neu.edu.cn (L.L.); 2401456@stu.neu.edu.cn (Y.F.); zhangying@mail.neu.edu.cn (Y.Z.); 2 CAS Key Laboratory of Forest Ecology and Silviculture, Institute of Applied Ecology, Chinese Academy of Sciences, Shenyang 110016, China

**Keywords:** *Echinacea purpurea*, biochar incorporation, multi-omics, phenylpropanoid pathway, medicinal compounds

## Abstract

*Echinacea purpurea* (L.) Moench is a medicinally valuable plant with well-documented pharmacological properties; however, its physiological and molecular responses to biochar amendment remain largely unexplored. This study applied integrated transcriptomic and metabolomic approaches to investigate the underlying mechanisms of biochar-induced effects in *E. purpurea*. Biochar amendment significantly promoted plant growth and improved nutrient status. Concurrent transcriptomic analysis revealed the molecular basis for these changes, identifying 4733 differentially expressed genes (DEGs). Further analysis showed significant enrichment in plant hormone signal transduction pathways, particularly those of auxin and jasmonic acid (JA). The activation of the JA pathway was specifically validated by the concurrent upregulation of its biosynthetic and metabolic genes, together with the marked accumulation of JA, jasmonoyl-isoleucine, and 12-hydroxy-jasmonoyl-isoleucine. Metabolomic profiling further revealed a pronounced upregulation of phenylpropanoid pathway metabolites, including 3-hydroxy-4-methoxycinnamic acid, chlorogenic acid methyl ester, ferulic acid O-hexoside, and coumarin derivatives such as 7-methoxy-4-methylcoumarin. Correlation analysis of transcriptomic and metabolomic data confirmed the concurrent up-regulation of phenylpropanoid biosynthetic genes. These integrated results reveal the mechanistic basis through which biochar application simultaneously promotes growth and enhances the secondary metabolism of *E. purpurea* by coordinately activating phytohormone signaling and phenylpropanoid biosynthesis. These results establish the potential of biochar in enhancing *E. purpurea* cultivation, with future work needed to determine optimal application rates.

## 1. Introduction

*Echinacea purpurea* (L.) Moench, commonly known as purple coneflower, is a perennial herb of the Asteraceae family native to North America and represents one of the most widely cultivated and economically important medicinal plants globally [1,2]. This plant is traditionally used to treat respiratory ailments by stimulating the immune system and exhibits a wide spectrum of pharmacological effects, including anti-inflammatory, antioxidant, and antiviral properties [3,4]. Phytochemical studies have identified a diverse profile of bioactive compounds in *E. purpurea*, primarily phenolic compounds, which include phenolic acids (e.g., chicoric acid, caftaric acid, and chlorogenic acid) and flavonoids, alongside alkamides, polysaccharides, glycoproteins, and essential oils [5]. Notably, the phenolic acids alone can comprise 2.0–2.8% or more of the plant material [6]. Both phenolic acids and flavonoids are biosynthesized via the phenylpropanoid pathway [7]. As a pivotal biosynthetic route, the activation level of this pathway is a major determinant of the abundance of these valuable metabolites in *E. purpurea*, making it a key focus of phytochemical research.

Integrated transcriptomic and metabolomic analysis systematically connects gene expression profiles with metabolic phenotypes, thereby providing powerful mechanistic insights into the regulation of medicinal compound biosynthesis in plants [8,9,10]. This strategy has been successfully applied in several medicinal plants. For example, it revealed a metabolic shift from primary to secondary metabolism during seed maturation in *Nelumbo nucifera* (sacred lotus) [11], and in *Carthamus tinctorius* (safflower), it has elucidated key aspects of flavonoid biosynthesis, including the regulatory network of flower color transition and tissue-specific distribution [12,13,14]. Similarly, such multi-omics profiling in *Uncaria rhynchophylla* (cat’s claw) has helped construct the biosynthetic framework for tetracyclic oxindole alkaloids [15]. In the case of *E. purpurea*, existing research reveals complementary yet disconnected insights. A study integrating transcriptomic data with chemical profiling identified key regulators of phenolic biosynthesis [5], though its scope was limited to this specific metabolite class. Other efforts have employed single-omics approaches: ^1^H nuclear magnetic resonance-based metabolomics revealed geographical influences on metabolite content [16], while transcriptomics uncovered methyl jasmonate-responsive long non-coding RNAs [17]. While a recent study has elucidated how intrinsic factors (plant age and organ) regulate metabolite accumulation via multi-omics approaches [18], the potential for extrinsic agronomic practices to systemically reprogram the plant’s transcriptome and metabolome remains largely unexplored.

The large-scale cultivation of *E. purpurea* for economic purposes aims to achieve high-quality plant material while meeting the growing demand for sustainable agricultural practices that minimize environmental impact and chemical inputs. Biochar (BC), a carbon-rich porous material, has been widely used in agriculture for soil improvement and sustainable development [19,20]. Its stable structure, high carbon content, and strong water-holding capacity contribute to enhanced nutrient availability, improved soil enzyme activity, and increased microbial diversity [21,22]. Beyond general agricultural applications, biochar has shown promising results in medicinal plant cultivation. For example, in *Andrographis paniculata* (green chiretta), biochar amendment significantly improved both plant yield and the content of the bioactive lactone andrographolide [23]. Furthermore, biochar application enhanced the production of pharmacologically active compounds and antioxidant properties in multiple medicinal species, including *Bacopa monnieri* (waterhyssop) and *Withania somnifera* (ashwagandha), even under heavy metal stress [24]. In *Crocus sativus* (saffron), biochar was also found to significantly alter the profile of bioactive components by increasing picrocrocin content [25]. These collective findings support the potential of biochar as an effective soil amendment for boosting growth and steering the accumulation of medicinal compounds in specialized crops such as *E. purpurea*. Despite this potential, no study has specifically investigated how biochar orchestrates the growth and medicinal compound biosynthesis in *E. purpurea* at an integrated transcriptomic and metabolomic level.

To address this knowledge gap, this study employed an integrated transcriptomic and metabolomic approach to investigate the physiological and molecular responses of *E. purpurea* to biochar amendment. Our objectives were to: (1) evaluate the growth-promoting effects of biochar; (2) identify differentially accumulated metabolites (DAMs), with a focus on medicinal compounds produced from phenylpropanoid pathway; and (3) elucidate the coordinated regulatory mechanisms by integrating transcriptomic data to link differentially expressed genes (DEGs) with metabolic shifts. This research aims to provide a theoretical foundation for developing cultivation protocols that simultaneously enhance both yield and medicinal compound accumulation in *E. purpurea*.

## 2. Results

### 2.1. Plant Growth and Nutrient Accumulation

**Figure 1:**
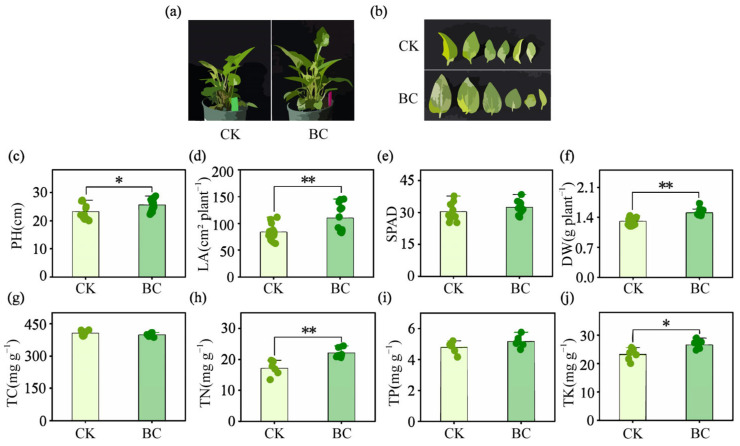
Plant growth and nutrient accumulation in *E. purpurea* under biochar amendment. (**a**,**b**) Visual phenotype of plants from the control (CK) and biochar-amended (BC) groups. (**c**–**j**) Bar graphs displaying the data for plant height (PH) (**c**), total leaf area (LA) (**d**), chlorophyll content (SPAD) (**e**), dry weight (DW) (**f**), total carbon (TC) (**g**), total nitrogen (TN) (**h**), total phosphorus (TP) (**i**), and total potassium (TK) (**j**). Data represent the mean ± SD (*n* = 9). Significant differences between BC and CK are denoted by asterisks (* *p* < 0.05, ** *p* < 0.01).

Compared with the control (CK), biochar application promoted visible growth enhancement in *E. purpurea*, as shown in the phenotypic images (Figure 1a,b). This was quantitatively supported by significant increases in plant height (PH, 10.38%), total leaf area (LA, 30.70%), and aboveground biomass (DW, 14.70%) compared to the control (Figure 1c,d,f; *p* < 0.05 for PH, *p* < 0.01 for LA and DW). Regarding leaf chlorophyll content, no significant difference was observed between the treatments (Figure 1e, *p* > 0.05). The biochar also differentially influenced plant nutrient content. Total nitrogen (TN) and total potassium (TK) were significantly increased by 28.96% (*p* < 0.01) and 14.55% (*p* < 0.05), respectively. In contrast, total carbon (TC) tended to decrease and total phosphorus (TP) tended to increase, but these changes were not statistically significant (*p* > 0.05).

### 2.2. Overview of RNA Sequencing, Assembly, and Functional Annotation

**Figure 2:**
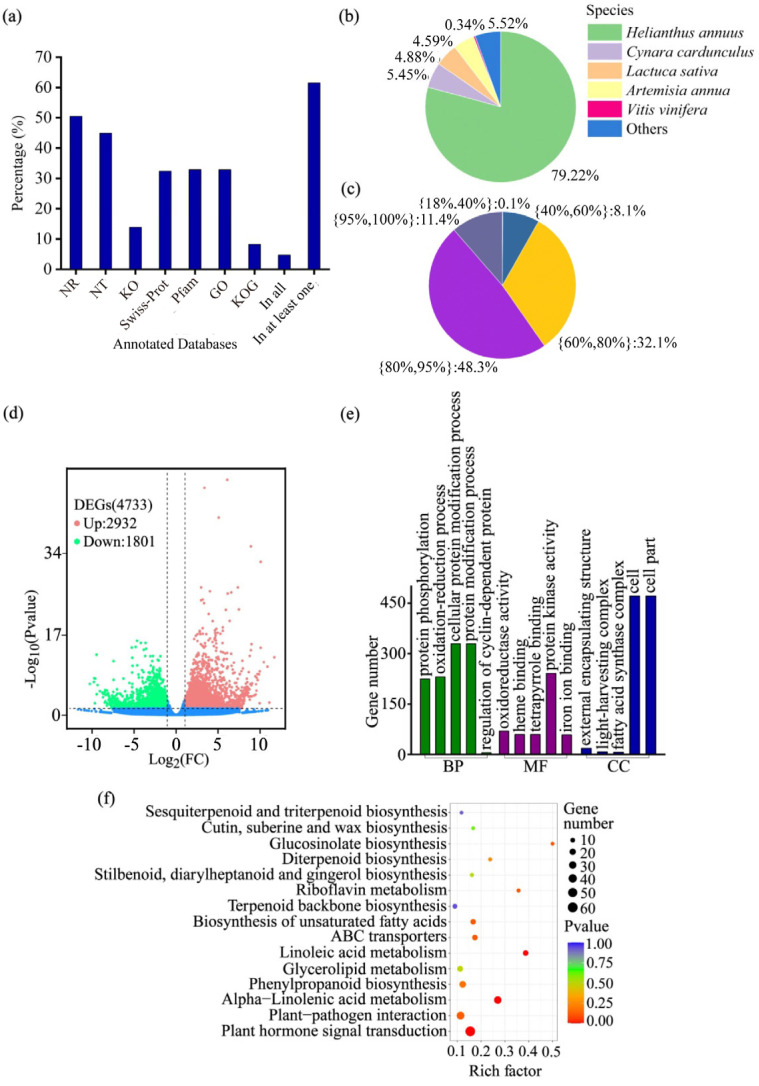
Transcriptomic profiling of *E. purpurea* leaves in response to biochar amendment. (**a**) Functional annotation statistics of unigenes across seven public databases. (**b**) Species distribution of homologous sequences. (**c**) Sequence similarity distribution of annotated unigenes. (**d**) Volcano plot identifying DEGs between biochar-amended and control groups. The red, green, and blue dots represent the up-regulated, down-regulated, and non-significant differentially expressed genes, respectively. (**e**) Gene Ontology (GO) functional classification of DEGs. (**f**) Kyoto Encyclopedia of Genes and Genomes (KEGG) pathway enrichment analysis of DEGs. Abbreviations in (**a**): NR, Non-Redundant Protein Sequence Database; NT, Nucleotide Sequence Database; KO, KEGG Orthology; Pfam, Protein Families Database; GO, Gene Ontology; KOG, euKaryotic Orthologous Groups.

A total of 277,002 transcripts were assembled from the clean reads, with an N50 length of 1609 bp and a mean length of 1143 bp. From these transcripts, 91,255 unigenes were generated, exhibiting an average length of 1051 bp and ranging from 301 bp to 16,585 bp. The length distributions of both transcripts and unigenes are detailed in Table S1. For a comprehensive functional annotation of the *E. purpurea* transcriptome, all assembled unigenes were aligned against seven public databases (NR, NT, KEGG, SwissProt, Pfam, GO, and KOG). Among the 91,255 unigenes, 56,161 (61.54%) were successfully annotated in at least one database (Figure 2a). Species distribution analysis of the annotated unigenes revealed that the majority exhibited the highest sequence similarity to *Helianthus annuus* (79.22%), followed by *Cynara cardunculus* (5.45%), *Lactuca sativa* (4.88%), *Artemisia annua* (4.59%), and *Vitis vinifera* (0.34%) (Figure 2b). Furthermore, of the total unigenes, 83,772 (91.8%) were successfully annotated against the databases with a sequence similarity ranging from 60% to 100% (Figure 2c). Based on this well-annotated transcriptome, we identified 4733 DEGs in response to biochar amendment, with 2932 up-regulated and 1801 down-regulated (Figure 2d). The prevalence of up-regulated genes suggests that biochar triggers an extensive transcriptional activation in *E. purpurea*. To decipher the functions of the identified DEGs, we conducted GO and KEGG enrichment analyses. In the GO analysis, terms for ‘protein modification process’ (biological process) and ‘protein kinase activity’ (molecular function) were significantly enriched (Figure 2e). In the KEGG analysis, significant enrichment was observed for key metabolic and signaling pathways, including ‘Plant hormone signal transduction’ (53 genes), ‘alpha-Linolenic acid metabolism’ (27 genes), and ‘Linoleic acid metabolism’ (12 genes) (Figure 2f).

### 2.3. Overview of Metabolomic Profiling and Differential Accumulation Metabolites

**Figure 3:**
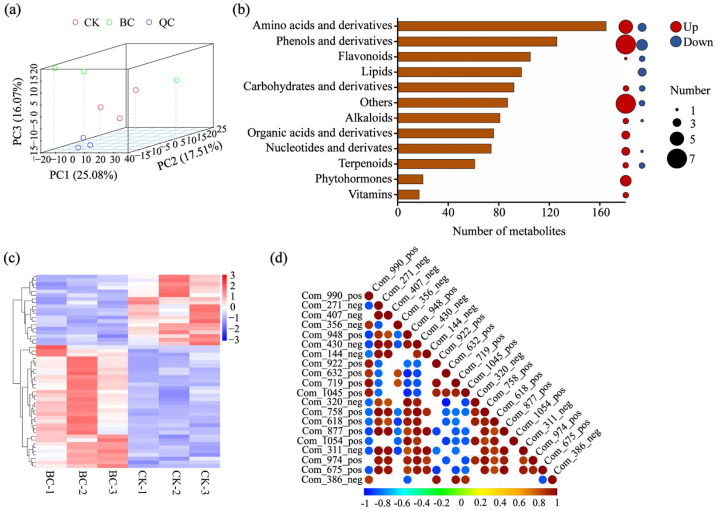
Metabolic profiling of *E. purpurea* leaves in response to biochar amendment. (**a**) Principal component analysis of all identified metabolites. (**b**) Classification of all identified metabolites and statistics of DAMs. (**c**) Hierarchical clustering analysis of all DAMs. (**d**) Correlation heatmap of the top 20 DAMs. In (**a**,**c**), CK represents the control group; BC, the biochar-amended group; QC, quality control sample.

Widely targeted metabolomic analysis was conducted to investigate the metabolic response of *E. purpurea* to biochar amendment. Principal component analysis (PCA) of the 1002 identified metabolites showed clear separation between BC and CK groups, with tight clustering of quality control (QC) samples confirming high data reproducibility (Figure 3a). Among all metabolites, amino acids and derivatives (165), phenolic acids (126), and flavonoids (105) were the most abundant classes (Figure 3b). Comparative analysis identified 58 DAMs, among which phenolic acids/derivatives and amino acids/derivatives collectively accounted for 19 and were predominantly up-regulated (7 and 5 metabolites, respectively). Phytohormones constituted another significantly up-regulated category, with all 4 identified DAMs increasing in abundance (Figure 3c). Consistent with the PCA results, hierarchical clustering of the DAMs clearly segregated the CK and BC samples (Figure 3c), revealing a metabolic response dominated by the up-regulation of 39 metabolites, compared to only 19 that were down-regulated. Additionally, correlation analysis of the top 20 DAMs indicated coordinated regulation among these key metabolites in response to biochar amendment (Figure 3d).

### 2.4. Association Analysis of Metabolome and Transcriptome

**Figure 4:**
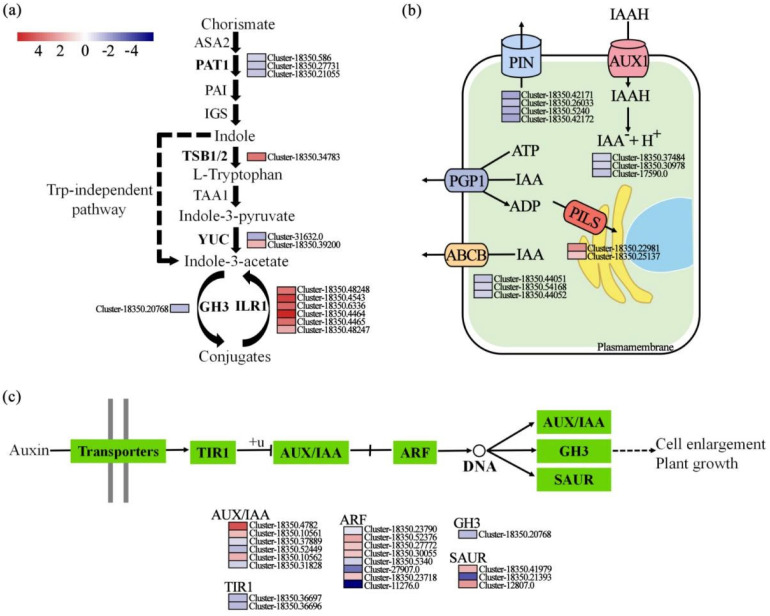
Auxin-related pathways in *E. purpurea* leaves in response to biochar amendment. Diagrams show auxin (**a**) biosynthesis, (**b**) transport, and (**c**) signal transduction. Gene expression heatmap (color bar: −4→0→4): Blue indicates low gene expression levels, while red indicates high expression levels (with 0 representing the baseline level); the colored blocks adjacent to the genes represent the relative expression levels of these genes across different samples. Abbreviations: *ASA2*, anthranilate synthase alpha 2; *PAI*, polyamide-imide; *TIR1*, transport inhibitor response 1; *TSB1/2*, tryptophan synthase beta 1/2; *TAA1*, tryptophan aminotransferase of Arabidopsis 1; *YUC*, YUCCA; *GH3*, Gretchen Hagen 3; *ARF*, auxin response factors; *ILR1*, IAA-leucine resistant 1; *PIN*, PIN-FORMED; *AUX1*, Auxin permease 1; *PGP1*, P-glycoprotein 1; *PILS*, PIN-LIKES; *ABCB*, ATP-binding cassette B; *SAUR*, small auxin up RNA.

KEGG enrichment analysis identified plant hormone signal transduction as a primary target of biochar, with 53 assigned DEGs (Table S2). The indole acetic acid (IAA) and jasmonic acid (JA) signaling pathways represented the most substantially altered sub-pathways, comprising 13 and 12 DEGs, respectively, and were accordingly prioritized for in-depth analysis. To gain a broader understanding of the auxin signaling events, we extended the analysis to upstream processes, including biosynthesis and the polar transport system. While a few genes associated with auxin biosynthesis, such as tryptophan synthase beta 1/2 (*TSB1/2*), were up-regulated, a profound reconfiguration of auxin homeostasis was revealed by the up-regulation of IAA-leucine resistant 1 (*ILR1*), most strikingly by a specific homolog (Gene ID: Cluster-18350.4464) with a log_2_FC of 6.76 (Figure 4a). Concurrently, transport dynamics were reshaped, as evidenced by the down-regulation of plasma membrane influx (Auxin permease 1 (*AUX1)*) and efflux (ATP-binding cassette B (*ABCB*), PIN-FORMED (*PIN*)) carriers, alongside the up-regulation of nuclear transport (PIN-LIKES (*PILS*)) genes (Figure 4b). Analysis of the IAA signal transduction pathway revealed a member-specific transcriptional reprogramming in response to biochar. Key changes included down-regulation of the auxin receptor gene transport inhibitor response 1 (*TIR1*) and the Gretchen Hagen 3 (*GH3*) conjugating enzymes, alongside member-specific expression shifts in downstream AUX/IAA repressors, auxin response factors (*ARF*) activators, and small auxin up RNA (*SAUR*) genes (Figure 4c). Collectively, these alterations define a modified auxin signaling network, thus offering a mechanistic insight into the biochar-induced growth enhancement.

**Figure 5:**
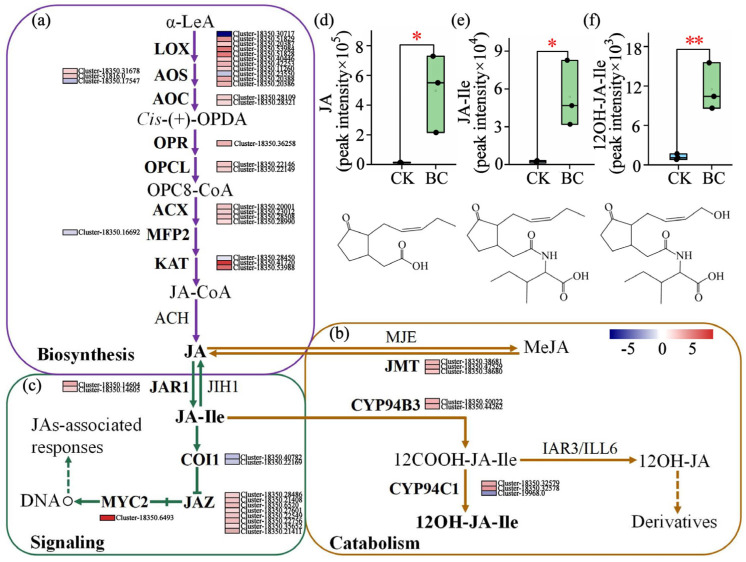
Gene expression and metabolite accumulation associated with the JA pathway in *E. purpurea* leaves in response to biochar amendment. Gene expression in the JA (**a**) biosynthesis, (**b**) metabolism, and (**c**) signaling pathways. (**d**–**f**) Accumulation levels of JA-related metabolites (JA (**d**), JA-Ile (**e**), and 12OH-JA-Ile (**f**)). Values are presented as mean ± SD (*n* = 3). Gene expression heatmap (color bar: −5→0→5): Blue indicates low gene expression levels, while red indicates high expression levels (with 0 representing the baseline level); the colored blocks adjacent to the genes represent the relative expression levels of these genes across different samples. Solid arrows indicate direct enzyme-catalyzed biochemical conversion steps; dashed arrows rep-resent regulatory/associative interactions. Asterisks indicate significant differences compared to the control: * *p* < 0.05, ** *p* < 0.01. Scheme of JA biosynthesis and catabolism made based on the works Huang et al. (2017) [26] and Delgado et al. (2021) [27]. Abbreviations: α-LeA, α-Linolenic acid; *LOX*, Lipoxygenase; *AOS*, Allene oxide synthase; *AOC*, Allene oxide cyclase; *OPDA*, 12-oxo-phytodienoic acid; *OPR*, OPDA reductase; *OPCL*, OPC-8:0 CoA ligase; *ACX*, Acyl-CoA oxidase; *MFP2*, Multifunctional protein 2; *KAT*, 3-ketoacyl-CoA thiolase; JA-CoA, Jasmonoyl-CoA; JA, Jasmonic acid; *JAR1*, Jasmonate resistant 1; JA-Ile, Jasmonoyl-L-isoleucine; MeJA, Methyl jasmonate; *JMT*, Jasmonate methyltransferase; *COI1*, Coronatine insensitive 1; *JAZ*, Jasmonate ZIM-domain protein; *MYC2*, MYC2 transcription factor; *IAR3/ILL6*, IAA-Ala Resistant 3/IAA-Leu Resistant-Like 6; *CYP94B3*, Cytochrome P450 94B3; *CYP94C1*, Cytochrome P450 94C1; *CYP94B3/C1*, Cytochrome P450 94B3/94C1.

To elucidate the JA signaling events, we investigated its upstream processes, including biosynthesis and metabolism. Analysis of JA biosynthesis (from α-Linolenic acid (α-LeA)) revealed a predominant up-regulation of genes (23 out of 28), including allene oxide cyclase (*AOC*), 12-oxo-phytodienoic acid reductase (*OPR*), OPC-8:0 CoA ligase (*OPCL*), acyl-CoA oxidase (*ACX*), and most isoforms of lipoxygenase (*LOX*), allene oxide synthase (*AOS*), and 3-ketoacyl-CoA thiolase (*KAT*), suggesting a potential shift in metabolic flux toward JA production (Figure 5a). This transcriptional activation extended to JA catabolism. Specifically, we observed up-regulation of the genes encoding jasmonate methyl esterase (JAM), which converts methyl jasmonate (MeJA) to JA, and Cytochrome P450 94B3 (*CYP94B3*), which converts JA-Ile to 12-carboxy-jasmonoyl-isoleucine (12COOH-JA-Ile). This was accompanied by predominant up-regulation of Cytochrome P450 94C1 (*CYP94C1*) isoforms, which further metabolize 12COOH-JA-Ile to 12-hydroxy-jasmonoyl-isoleucine (12OH-JA-Ile). This collective gene expression profile indicates a channeling of metabolic flux that favors the accumulation of JA, JA-Ile, and 12OH-JA-Ile (Figure 5b). At the signaling level, the pathway was modulated through a two-tiered regulatory mechanism: a negative feedback circuit—initiated by up-regulation of Jasmonate resistant 1 (*JAR1*), characterized by Jasmonate ZIM-domain protein (*JAZ*) up-regulation and Coronatine insensitive 1 (*COI1*) down-regulation—that releases inhibition on MYC2 transcription factor (*MYC2*), complemented by a direct up-regulation of *MYC2* itself, which synergistically enhances the output of the entire cascade (Figure 5c). Consistent with this transcriptional reprogramming, biochar treatment ultimately induced dramatic increases in the levels of JA (41.62-fold), JA-Ile (9.66-fold), and 12OH-JA-Ile (24.07-fold) compared to the control (*p* < 0.05 for JA and JA-Ile; *p* < 0.01 for 12OH-JA-Ile) (Figure 5d–f). The synergistic enrichment of the “Plant hormone signal transduction” pathway by both DEGs and DAMs (Figure S2), to which JA-related components were major contributors, provides systematic, multi-omics validation for the coordinated activation of the JA pathway.

**Figure 6:**
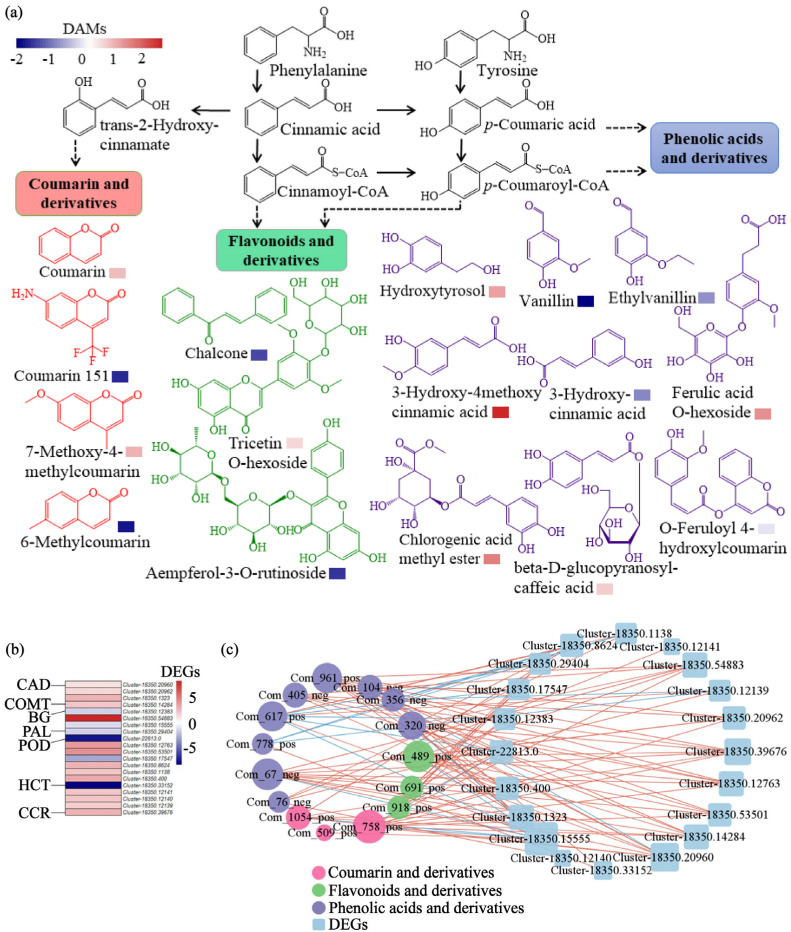
Metabolite accumulation and gene expression associated with the phenylpropanoid pathway in *E. purpurea* leaves in response to biochar amendment. (**a**) Accumulation levels of key phenylpropanoid-related metabolites. (**b**) Expression of genes involving in phenylpropanoid pathway. (**c**) Correlation network between DAMs and DEGs. In a, the identified DAMs with increased level were categorized into three classes. In (**c**), red or blue edges represent positive or negative correlations, respectively. Abbreviations: *CAD*, cinnamyl alcohol dehydrogenase; *COMT*, caffeic acid O-methyltransferase; *BG*, beta-glucosidase; *PAL*, phenylalanine ammonia-lyase; *POD*, peroxidase; *HCT*, shikimate O-hydroxycinnamoyltransferase; *CCR*, cinnamoyl-CoA reductase. Solid arrows denote single-step enzymatically catalyzed direct metabolic reactions; dashed arrows denote the simplified representation of multiple consecutive metabolic reactions.

Given that phenolic acids and flavonoids (total 231 compounds) constituted the most abundant metabolite classes and are primarily synthesized via the phenylpropanoid pathway, we focused on this pathway and evaluated its core metabolites and genes. We classified the 16 DAMs into three groups (Figure 6a): flavonoids and derivatives (chalcone, tricetin O-hexoside, aempferol-3-O-rutinoside), coumarins and derivatives (coumarin, coumarin 151, 7-methoxy-4-methylcoumarin, 6-methylcoumarin), and phenolic acids and derivatives (hydroxytyrosol, ethylvanillin, vanillin, 3-hydroxy-4methoxy cinnamic acid, 3-hydroxy-cinnamic acid, ferulic acid O-hexoside, chlorogenic acid methyl ester, beta-D-glucopyranosyl-caffeic acid, acid O-feruloyl 4-hydroxylcoumarin). Among them, the most pronounced accumulation was observed for 3-hydroxy-4-methoxycinnamic acid (log_2_FC = 2.64), followed by chlorogenic acid methyl ester (log_2_FC = 1.63), ferulic acid O-hexoside (log_2_FC = 1.45), hydroxytyrosol (log_2_FC = 1.26), 7-methoxy-4-methylcoumarin (log_2_FC = 1.12), coumarin (log_2_FC = 0.97), β-D-glucopyranosyl-caffeic acid (log_2_FC = 0.78), and tricetin O-hexoside (log_2_FC = 0.69). O-feruloyl 4-hydroxycoumarin showed a minimal increase (log_2_FC = 0.58). In contrast, several metabolites showed a marked reduction: vanillin (log_2_FC = −2.08), 6-methylcoumarin (log_2_FC = −1.80), coumarin 151 (log_2_FC = −1.69), chalcone (log_2_FC = −1.38), and ethylvanillin (log_2_FC = −0.67). Within the 20 DEGs identified in the phenylpropanoid pathway, 14 were up-regulated (Figure 6b). Key biosynthetic gene families, including cinnamyl alcohol dehydrogenase (*CAD*), caffeic acid O-methyltransferase (*COMT*), and cinnamoyl-CoA reductase (*CCR*), exhibited uniform up-regulation across all members (log_2_FC 1.09–2.59, 2.04, and 2.41, respectively). Other families, such as beta-glucosidase (*BG*), peroxidase (*POD*), and shikimate O-hydroxycinnamoyltransferase (*HCT*), showed a predominant up-regulation, with one BG gene displaying the most pronounced induction (log_2_FC = 7.76). To elucidate the functional relationships within the phenylpropanoid pathway, we constructed a correlation network between the 20 DEGs and 16 DAMs, which revealed extensive positive associations (Figure 6c). This coordinated regulation was highlighted by two key aspects: pivotal metabolites served as hubs, correlating with multiple genes, for instance, chlorogenic acid methyl ester showed significant positive correlations with seven genes, including those encoding key enzymes such as *CCR*, *COMT*, *CAD*, and *POD*, and similarly, 7-methoxy-4-methylcoumarin was correlated with nine genes, encompassing the same core enzymatic families. Concurrently, key genes functioned as central nodes, associating with numerous metabolites. This included the *BG* gene family, which correlated with 13 of the 16 metabolites, and the *CAD* gene family, associated with nine metabolites. The extensive interconnectivity within this co-expression network demonstrates a tightly coordinated transcriptional and metabolic reprogramming of the phenylpropanoid pathway, identifying it as a central component of the biochar effects in *E. purpurea*.

**Figure 7:**
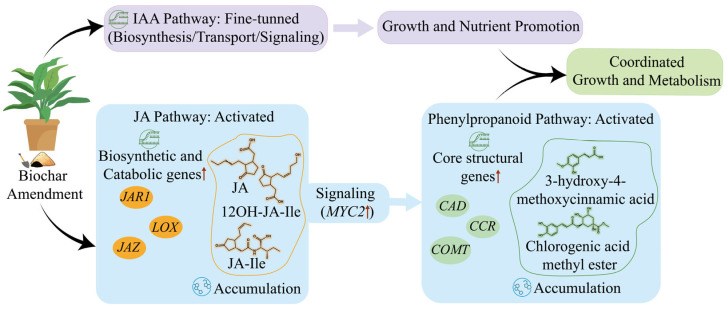
Proposed model for biochar-induced coordination of growth and secondary metabolism in *E. purpurea*. The biochar-induced improvements in plant growth and nutrient status are underpinned by a widespread transcriptional reprogramming, involving the fine-tuning of the auxin pathway. This is coupled with a strong activation of JA pathway, which is evidenced by the upregulation of biosynthetic genes and the marked accumulation of JA, jasmonoyl-isoleucine, and 12-hydroxy-jasmonoyl-isoleucine, leading to the upregulation of the key transcription factor *MYC2*. *MYC2* subsequently activates the phenylpropanoid pathway, inducing biosynthetic genes and resulting in the accumulation of specific phenolic compounds, such as 3 hydroxy 4 methoxycinnamic acid and chlorogenic acid methyl ester. This coordinated interplay ultimately enables the simultaneous enhancement of plant growth and secondary metabolism. Black arrows indicate the direction of process flow; red upward arrows denote upregulated genes; blue/pink arrows represent directional connections between functional modules.

Based on these integrated findings, we propose a working model that delineates how biochar coordinately activates phytohormone signaling to orchestrate both growth promotion and phenylpropanoid metabolism (Figure 7).

## 3. Discussion

### 3.1. Growth Enhancement and Transcriptomic Reprogramming in E. purpurea Mediated by Biochar

Biochar, as an efficient soil amendment, has been widely documented in agricultural systems to enhance crop growth and yield through multiple mechanisms, including improved soil physical structure, increased nutrient availability, and modulation of microbial communities [28,29,30]. Consistent with these findings, our study demonstrates that biochar amendment significantly promoted the growth of the medicinal plant *E. purpurea*, as reflected by increases in PH, leaf area, and dry biomass (Figure 1a–f). This growth-promoting effect is further supported by studies in other medicinal plants, such as *Andrographis paniculata*, *Withania somnifera*, and *Alpinia zerumbet*, where biochar application enhanced biomass accumulation and photosynthetic performance [24,25], underscoring its broad potential in medicinal plant cultivation. In addition to growth phenotypes, biochar significantly increased leaf nitrogen and potassium content in *E. purpurea* (Figure 1h,j), suggesting that improved nutrient status constitutes a key physiological basis for growth promotion [31]. These benefits are largely attributed to the integrated improvement of soil physicochemical properties by biochar, particularly in optimizing the availability and uptake of key elements such as nitrogen and phosphorus [32,33,34]. Moreover, biochar has been shown to enhance nitrogen retention and utilization efficiency in the soil–plant system by reducing nitrogen losses via adsorption and promoting microbial-mediated nitrogen transformation processes [35,36,37]. Collectively, these results demonstrate that biochar amendment led to enhanced growth of *E. purpurea*, along with a systematic improvement in plant nutritional status, suggesting a potential functional link between these two aspects.

Transcriptomic analysis revealed that biochar application induced a systemic reprogramming of gene expression in *E. purpurea* (Figure 2d). The DEGs showed significant enrichment in plant hormone signal transduction pathways, particularly in the auxin signaling pathway where the most pronounced alterations were observed （Figure 2f, Figure 4 and Figure 5, Table S2). This transcriptional reprogramming suggests that biochar’s impact on *E. purpurea* involves not only enhanced nutrient acquisition but also the modulation of intrinsic developmental signaling networks. This finding is consistent with the model proposed by Viger et al., in which biochar promotes plant growth primarily by activating an auxin-dominated hormonal network. Their study further demonstrated that this process involves the upregulation of cell wall loosening-related genes and enhanced expression of transporters for water and nutrients, collectively facilitating cell expansion and resource acquisition [38]. Our earlier work in maize demonstrated that biochar and straw return improve root system architecture by upregulating key genes involved in cell division and expansion [39]. Similarly, Wang et al. (2022) [40] observed a broad activation of hormone signaling pathways under straw return, with auxin-related genes exhibiting particularly strong induction. These independent reports suggest that hormone reprogramming, especially auxin pathway activation, is a conserved mechanism for growth promotion mediated by exogenous carbon inputs.

In the present study, biochar amendment markedly altered the expression of genes involved in auxin metabolism and transport (Figure 4a). Key observations include the suppression of most genes in the tryptophan-dependent IAA biosynthesis pathway, coupled with a significant upregulation of *ILR1*—which releases active free IAA from conjugated forms—and the downregulation of *GH3* homologs that promote IAA conjugation (Figure 4a). This expression profile suggests that biochar enhances the conversion of conjugated IAA pools into the active free form, thereby rapidly increasing local auxin levels, a mechanism supported by existing literature on conjugated IAA as a reversible storage pool [41,42]. Furthermore, we found that key polar auxin transport carriers (e.g., *AUX1*, *ABCB*, and *PIN*) were generally downregulated, whereas genes of the *PILS* family, which facilitate IAA sequestration into the endoplasmic reticulum, were upregulated (Figure 4b). Previous studies have established that PILS proteins negatively regulate nuclear auxin signaling by confining auxin to the endoplasmic reticulum [43,44]. Together, these findings indicate that biochar reshapes auxin homeostasis not by boosting its synthesis, but by promoting local activation, restricting long-distance movement, and altering its subcellular distribution. The refined pattern of auxin observed in *E. purpurea* leaves may thus be integral to biochar-mediated growth enhancement.

### 3.2. Enrichment of Secondary Metabolites and Activation of Jasmonic Acid Signaling and Phenylpropanoid Biosynthesis in E. purpurea Mediated by Biochar

The pharmacological activity of medicinal plants is closely linked to the content and profile of their secondary metabolites [45]. As an important medicinal species, *E. purpurea* exhibits immunomodulatory and anti-inflammatory properties primarily attributed to characteristic secondary metabolites such as caffeic acid derivatives, flavonoids, and alkamides [2,18]. This study demonstrates that biochar treatment significantly enhanced the accumulation of various phenolic and coumarin compounds in the leaves of *E. purpurea*, including chlorogenic acid methyl ester and 3-hydroxy-4-methoxycinnamic acid (Figure 3b and Figure 6). Nigam et al. (2021) [24] reported that even under lead-cadmium co-contamination stress, biochar could markedly increase total phenolic and flavonoid contents in several medicinal species such as *Andrographis paniculata* and *Withania somnifera*, while also enhancing their antioxidant activity. Similarly, Zulfiqar et al. (2021) [46] confirmed in *Alpinia zerumbet* that biochar effectively promoted metabolite synthesis and alleviated oxidative stress. These consistent findings suggest that biochar possesses cross-species potential and reliability in regulating secondary metabolism in medicinal plants.

Our study indicated that biochar amendment activated the JA signaling pathway in *E. purpurea* leaves, as evidenced by the marked accumulation of bioactive JA-Ile and its metabolite 12OH-JA-Ile, along with the reprogramming of key pathway genes, including the significant upregulation of *JAR1*, a gene responsible for synthesizing active JA-Ile (Figure 5b,c). As a central signaling molecule, JA-Ile promotes the formation of the COI1–JAZ co-receptor complex, thereby triggering the ubiquitination and degradation of JAZ repressor proteins, which in turn derepresses transcription factors like MYC and initiates downstream secondary metabolic programs [47,48,49]. JAs are considered key hubs linking environmental stimuli to the regulation of secondary metabolism [17,50,51]. This mechanism has been validated in multiple medicinal plants, where JA signaling activation effectively stimulates the synthesis of characteristic metabolites, including ginsenosides in *Panax ginseng* [52], artemisinin in *Artemisia annua* [53], and taxol in *Taxus* species [54]. In *E. purpurea*, Tahmasebi et al. (2019) [5] similarly demonstrated that MeJA treatment significantly increased the content of phenolic compounds including caffeic acid and chlorogenic acid, as well as phenylalanine ammonia-lyase (PAL) activity, further supporting the close relationship between JA signaling and secondary metabolite accumulation in this species. Moreover, in this study, the concurrent upregulation of genes responsible for JA-Ile oxidative inactivation (*CYP94B3/CYP94C1*) and the negative regulator *JAZ*, together with the accumulation of 12OH-JA-Ile, formed a finely tuned negative feedback loop (Figure 5). This suggests that biochar-induced JA signaling is a tightly controlled, transient activation event, one that effectively initiates secondary metabolism while avoiding potential growth inhibition caused by sustained high JA levels, reflecting a sophisticated trade-off between growth promotion and enhanced defense/metabolism in plants [55,56].

In line with this view of JA’s broad regulatory role, a potential cross-talk likely exists between the JA signaling and phenylpropanoid pathways. Evidence suggests that the JA-activated transcription factor MYC may regulate key phenylpropanoid genes by binding to specific cis-elements in their promoters, thereby influencing metabolic flux [57,58]. This is supported by findings in the medicinal plant *Salvia miltiorrhiza*, where JA-responsive *MYC2* directly enhances phenolic acid biosynthesis [59]. Moreover, a similar induction was observed in tea plants, where organic fertilizer application concurrently activated the JA-related α-linolenic acid metabolism and upregulated phenylpropanoid pathway genes (e.g., *PAL*, *4CL*, *FLS*) [60]. In the present study, the overall upregulation of phenylpropanoid pathway genes coincided with the activation of JA signaling (Figure 6), suggesting that biochar may influence the expression of structural genes in phenylpropanoid metabolism via JA signaling.

The phenylpropanoid pathway is recognized as a central route for the synthesis of phenolic acids, coumarins, and flavonoids in plants [6]. Our transcriptomic data revealed a general upregulation trend of key phenylpropanoid genes, such as *CAD*, *COMT*, and *CCR*, in *E. purpurea* following biochar amendment (Figure 6). Further correlation analysis showed that certain metabolites, including chlorogenic acid methyl ester, were positively correlated with seven genes, while 7-methoxy-4-methylcoumarin was associated with nine genes. This co-expression pattern implies that biochar treatment is linked to the remodeling of the phenylpropanoid metabolic network, potentially by influencing the operational state of the plant’s metabolic system, ultimately enhancing specific secondary metabolite synthesis pathways. Activation of the phenylpropanoid pathway is commonly associated with plant stress responses, and its phenolic products are known to play roles in antioxidant processes [7]. In addition, appropriate soil nutrient management, such as rational fertilization, has been shown to promote the synthesis and accumulation of phenylpropanoids and flavonoids by modulating metabolic balance in plants [61,62]. The enhanced uptake of nitrogen and potassium mediated by biochar (Figure 1) likely improved the plant’s nutritional status, creating favorable conditions for phenylpropanoid-based secondary metabolism. Looking beyond direct nutrition, the multi-omics foundation established here paves the way for future investigations into biochar-mediated rhizosphere processes [63], which represent a promising direction for understanding the full scope of its beneficial effects. Collectively, our study underscores the power of integrated multi-omics analysis in deciphering complex plant responses to agronomic practice. By applying this strategy, previously exemplified in studies of intrinsic regulation [11,18,64], to the underexplored domain of extrinsic soil management, we not only reveal how biochar coordinately reprograms the transcriptome and metabolome but also establish a mechanistic framework for understanding soil–plant interactions in medicinal plant cultivation.

## 4. Materials and Methods

### 4.1. Soil Preparation, Experimental Design, and Plant Cultivation

The soil used in this experiment was collected from the top 20 cm layer of a farm in Shenyang City, Liaoning Province, China. Following collection, the soil was air-dried, homogenized, and sieved to remove stones and plant residues. Its basic physicochemical properties were as follows: pH 5.85; total nitrogen, 1.69 g·kg^−1^; total carbon, 16.76 g·kg^−1^; total phosphorus, 0.437 g·kg^−1^; total potassium, 1.78 g·kg^−1^; ammonium nitrogen, 3.21 mg·kg^−1^; available phosphorus, 19.49 mg·kg^−1^; and available potassium, 153.1 mg·kg^−1^. The processed soil was filled into 1.5 L pots, with each pot containing 1.2 kg of soil (dry weight). The experiment comprised a biochar treatment and a control without biochar. Biochar was thoroughly mixed with the potting soil at a rate of 1.3% (*w*/*w*). This dosage was determined based on local agronomic practice and preliminary experiments. The biochar, supplied by the Jinhefu Agriculture Development Company (Shenyang, China), was pyrolyzed from maize straw at 450–500 °C and had the following characteristics: pH 8.10; organic matter 0.307 g·kg^−1^; total nitrogen 13.97 g·kg^−1^; total phosphorus 2.24 g·kg^−1^; and total potassium 34.55 g·kg^−1^. Uniform two-month-old *E. purpurea* seedlings with similar appearances, plant height, and leaf quantity were selected to ensure genetic and physiological homogeneity at the start of the experiment, thereby minimizing pre-existing variation and allowing a clearer attribution of observed effects to the biochar treatment. After transplanting, the pots (twelve replicates per treatment) were randomly arranged in the growth chamber to minimize positional effects and cultivated under a 16/8 h light/dark cycle at 18–27 °C., following the method described in Sun et al. (2023) [65]. Each pot was irrigated regularly to avoid excessive drainage. After 2 months biochar incorporation, *E. purpurea* plants were collected. Then, the plants were divided into two parallel groups: one for growth measurement (nine biological replicates) and another for omics analysis. For transcriptome and metabolome analysis, three biological replicates per treatment were used. Each replicate for omics was an independent plant sample. After collection, the leaf samples were immediately frozen and then stored at −80 °C until subsequent metabolite identification, transcriptome sequencing, and quantitative real-time polymerase chain reaction (qRT-PCR) validation.

### 4.2. Growth and Elemental Composition Measurement

PH, LA, and SPAD were measured in accordance with the method of Sun et al. (2023) [65]. Afterward, the shoot DW was determined via oven-drying at 80 °C until constant weight. TC and TN contents were quantified using elemental analysis (vario MACRO cube, Elementar Analysensysteme GmbH, Langenselbold, Germany). For TP determination, dried root samples were digested in a high-pressure microwave system (MWD-700, Shanghai Sineo Microwave Chemistry Technology Co., Ltd., Shanghai, China) with HF:H_2_O_2_:H_2_SO_4_ (3:3:7 mL) at 200 °C (15 min ramp, 20 min hold). The digestate was diluted to 50 mL with deionized water containing 2,4-dinitrophenol indicator (100 μL) and analyzed via the molybdenum-blue method at 880 nm using ascorbic acid and potassium antimonyl tartrate. TK was determined by acid digestion and subsequent analysis with flame atomic emission spectrometry.

### 4.3. Transcriptomic Sequencing and qRT-PCR

RNA isolation was carried out following a method described in our previous work [40] and RNA-seq was carried out using an Illumina HiSeq 6000 system (Illumina, San Diego, CA, USA) at Novogene Biotech (Beijing, China). The sequenced reads (NCBI SRA accession number: PRJNA1346659) were filtered by the removal of low-quality reads for subsequent analysis. Transcriptome assembly sequences were annotated by seven databases, including NR, NT, KO, Swiss-Prot, Pfam, KOG, and GO. DEGs were identified at a cutoff of |(Log_2_FC)| >1 (false discovery rate and adjusted *p* < 0.05). The identified DEGs were subsequently subjected to functional enrichment analyses, including KEGG pathway and GO analyses. Ten DEGs with different putative functions were selected to confirm the RNA-Seq results by performing qRT-PCR following our previous work [40]. The correlation between RNA-seq and qRT-PCR data is presented in Figure S1.

### 4.4. Metabolomic Analysis

The widely targeted metabolomic analysis was performed using the high-performance liquid chromatography tandem mass spectrometry with an ExionLC™ AD system and an Xselect HSS T3 column (150 × 2.1 mm^2^, 2.5 μm) coupled to a QTRAP^®^ 6500+ mass spectrometer (SCIEX) at Novogene Biotech (Beijing, China) following a method described in our previous work [40]. Metabolite identification adhered to the Metabolomics Standards Initiative (MSI) guidelines, primarily achieving Level 2 confidence based on the matching of high-resolution MS/MS spectraThe data files were processed using SCIEX OS software (v. 1.4, SCIEX, Shanghai, China) to integrate and correct peaks, and metabolites were identified by searching the Novogene in-house database. After that, the DAMs were screened using the partial least squares-discriminant analysis with thresholds of variable importance in the projection > 1.0, *p*-value < 0.05, and FC > 1.2 or <0.833. The complete processed metabolomics dataset generated during this study is provided in the Supplementary Materials (Supplementary Dataset S1).

### 4.5. Data and Statistical Analysis

The effects of biochar treatment on growth and nutrient parameters were assessed using one-way analysis of variance (ANOVA) in SPSS 25.0 (IBM Corp., Armonk, NY, USA). In transcriptomic analysis, DEGs were identified through volcano plot filtering based on −Log_10_ (*p*-value). PCA was performed using the Vegan package (v. 2.5-7, R Foundation for Statistical Computing, Vienna, Austria) and heatmaps were generated using the pheatmap package (v.1.0.12, R Foundation for Statistical Computing, Vienna, Austria) to illustrate sample segregation and the expression profiles of DEGs or DAMs, respectively.

## 5. Conclusions

In summary, this integrated transcriptomic and metabolomic study elucidates the physiological and molecular mechanisms by which biochar amendment enhances both the biomass and the production of medicinal compounds in *E. purpurea*. The observed growth promotion is associated with improved plant nutrient status and extensive reprogramming of phytohormone signaling pathways, most notably a massive activation of the JA pathway. Concurrently, the marked accumulation of key medicinal phenylpropanoids, such as chlorogenic acid derivatives and coumarins, is directly supported by the coordinated up-regulation of their corresponding biosynthetic genes, providing a multi-omics validation for the enhanced synthesis of these valuable metabolites. From a practical perspective, the simultaneous increase in both biomass and high-value medicinal metabolites highlights the potential of biochar as a sustainable soil management strategy for the commercial cultivation of *E. purpurea*. While this study demonstrates the positive effects of biochar amendment under controlled conditions, future research should now determine the optimal dosage, evaluate diverse biochar types, and validate these benefits under field conditions to fully assess its agronomic potential.

## Data Availability

Sequence data from this work can be found in the NCBI database (PRJNA1346659).

## Supplementary Materials

The following supporting information can be downloaded at https://www.mdpi.com/article/10.3390/ijms262311249/s1.
