# Supplementary material for: Integrated Transcriptomic and Metabolomic Analysis Reveals Biochar-Induced Enhancement of Growth and Secondary Metabolism in the Medicinal Plant Echinacea purpurea
Source: Int J Mol Sci. 2025 Nov 21;26(23):11249. doi: 10.3390/ijms262311249 (PMC12691805; doi:10.3390/ijms262311249)
Supplement: Supplementary file 1 [file ijms-26-11249-s001.zip › ijms-3978596-supplementary.pdf]

Supplementary File

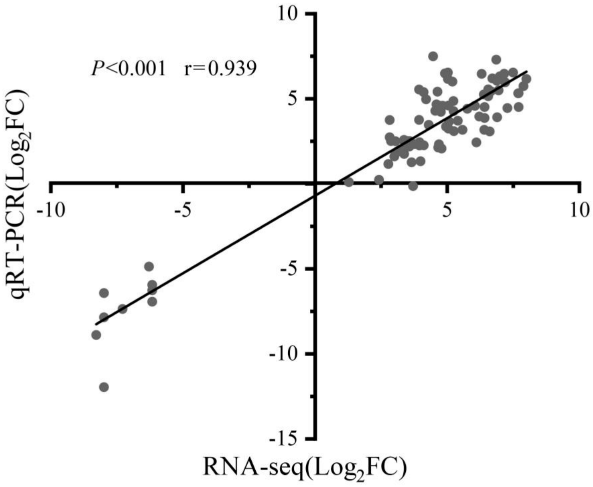

Figure S1. Correlation between RNA-seq and qRT-PCR data. Each RNA-seq expression data was plotted against qRT-PCR data and fitted into a linear regression.

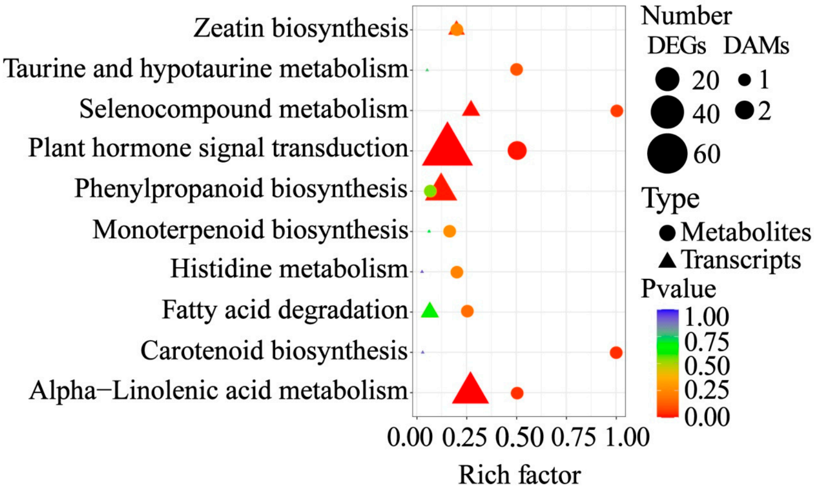

Figure S2. Joint KEGG pathway enrichment analysis of transcriptomic and metabolomic data. The scatter plot displays pathways significantly enriched by both differentially expressed genes (DEGs) and differentially accumulated metabolites (DAMs) following biochar amendment. The X-axis represents the rich factor (the proportion of DEGs/DAMs annotated in a given pathway relative to all annotated genes/metabolites), and the Y-axis shows the KEGG pathways. The size of the points reflects the number of DEGs or DAMs mapped to the respective pathway.

Table S1 Number and length distribution of the transcripts and unigenes from RNA-seq

	Total number	Total length	Average length (bp)	N50 length (bp)	N90 length (bp)	Length range (bp)			
						300-500	500-1000	1000-2000	>2000
Transcript	277002	316613286	1143	1609	513	76541	87110	72229	41122
Unigene	91255	95909005	1051	1487	471	28484	31465	19757	11549

Table S2 List of DEGs involved in plant hormone signal transduction

Plant Hormone	Gene Description (Symbol)	Number
Auxin (IAA)	Auxin1(AUX1)	3
	Auxin/indole-3-acetic acid (AUX/IAA)	6
	Auxin response factor (ARF)	8
	Gretchen hagen 3 (GH3)	1
	Small auxin-up RNA (SAUR)	3
	Transport inhibitor response 1 (TIR1)	2
Brassinosteroid (BR)	Cyclin D3 (CYCD3)	2
	Brassinosteroid insensitive 1-associated receptor kinase 1 (BAK1)	1
	BRI1 kinase inhibitor 1 (BKI1)	1
	Protein brassinosteroid insensitive 2 (BIN2)	1
Jasmonic acid (JA)	Jasmonate ZIM domain-containing protein (JAZ)	8
	Coronatine-insensitive protein 1 (COI-1)	2
	Jasmonic acid-amino synthetase (JAR1)	1
Gibberellin (GA)	Gibberellin Insensitive Dwarf 2 (GID2)	1
	Gibberellin Insensitive Dwarf 1 (GID1)	2
	DELLA protein (DELLA)	1
Cytokinine (CTK)	Histidine-containing phosphotransfer peotei (AHP)	1
	Two-component response regulator ARR-A family (ARR-A)	2
	Two-component response regulator ARR-B family (ARR-B)	1
	Arabidopsis histidine kinase 2/3/4 (AHK2/3/4)	4
Abscisic acid (ABA)	Serine/threonine-protein kinase (SRK2)	2
	Protein phosphatase 2C (PP2C)	2
	Abscisic acid receptor PYR/PYL family (PYL)	1
Ethylene (ETH)	Ethylene receptor (ETR)	1
	Ethylene-insensitive protein 3 (EIN3)	2
	Ethylene-responsive transcription factor 1 (ERF1)	1
Salicylic acid (SA)	Non-expressor of pathogenesis-related genes 1 (NPR1)	1
Phytochrome(PHY)	Phytochrome-interacting factor 3 (PIF3)	1
